# Studies on Kyphoplasty over 20 years by science mapping method: Kyphoplasty by science mapping method

**DOI:** 10.1097/MD.0000000000031179

**Published:** 2022-10-21

**Authors:** Serhat Comert

**Affiliations:** a Yildirim Beyazit University Yenimahalle Training and Research Hospital Department of Neurosurgery, Yenimahalle, Ankara, Turkey.

**Keywords:** bibliometric analysis, Kyphoplasty, scientific mapping, Web of Science Core Collection

## Abstract

This is a bibliometric analysis. Performed by Scientific Mapping technique. The purpose of the study is to analyze articles on Kyphoplasty. Kyphoplasty is a minimally invasive surgical method. Bibliometrics is the mathematical and statistical quantitative analysis of works. One of the primary uses of bibliometric analysis is scientific mapping.

**Method:**

To conduct the bibliometric analysis of the subject of Kyphoplasty, the Web of Science Core Collection database was preferred. After the selection of the data set, the data were filtered; as a result, the study was carried out on 2236 articles. Researchers, journals, articles, institutions, and the studies’ countries were analyzed.

**Results:**

According to the number of articles published in Kyphoplasty, China, USA, Germany, Korea, and Italy are among the leading countries. According to the analysis, the authors with the highest h-index value are Yang HL and Hirsch JA. The *European Spine Journal* and the *Spine* are the most impactful journals.

**Conclusions:**

Our study was carried out with the Science Mapping technique using Bibliometrics software. This type of work has become popular in recent years. Such studies are not common in the field of neurosurgery.

## 1. Introduction

Kyphoplasty is a minimally invasive surgical method used to treat spinal compression fractures. Compression fractures typically occur in the spine’s thoracic region, which includes the T1 to T12 vertebrae, but can also occur in the lumbar region, which includes the L1 to L5 vertebrae. Kyphoplasty aims to reduce pain from the fracture, stabilize the vertebrae and return it to its standard height.^[1,2]^

The amount of information in the literature is increasing rapidly. As knowledge increases, interdisciplinary relationships increase in parallel. The data that provide knowledge is becoming more complex every day. As a result, it becomes difficult to comprehend the research area structurally and determine its development and orientation. New methods are required to see the research area as a whole. Bibliometric methods evaluate authors, journals, and countries’ performance and research models. Moreover, bibliometric methods can be beneficial in determining the collaboration between them.^[3,4]^

Especially today, when the literature has become highly comprehensive, the importance of correct data analysis has increased. This is where bibliometric methods come into play since bibliometric methods are used to analyze large volumes of data in the literature. The analyzes contain information about a scientific discipline, the subject studied, academic institutions, countries, authors, and cooperation between authors.^[5–7]^

One of the primary uses of bibliometric analysis is scientific mapping, which aims to investigate and visualize the relationships of elements such as scientists, universities, and scientific works.^[8]^

Some software can be utilized for bibliometric applications. This software can be listed as follows Gephi, UCINET, Pajek, CoPalRed, Cytoscape, CiteSpace II, and VOSviewer.

The study’s primary purpose is to analyze articles on Kyphoplasty with the help of the science mapping technique.

## 2. Materials and Methods

### 2.1. Research workflow

**Figure 1.**
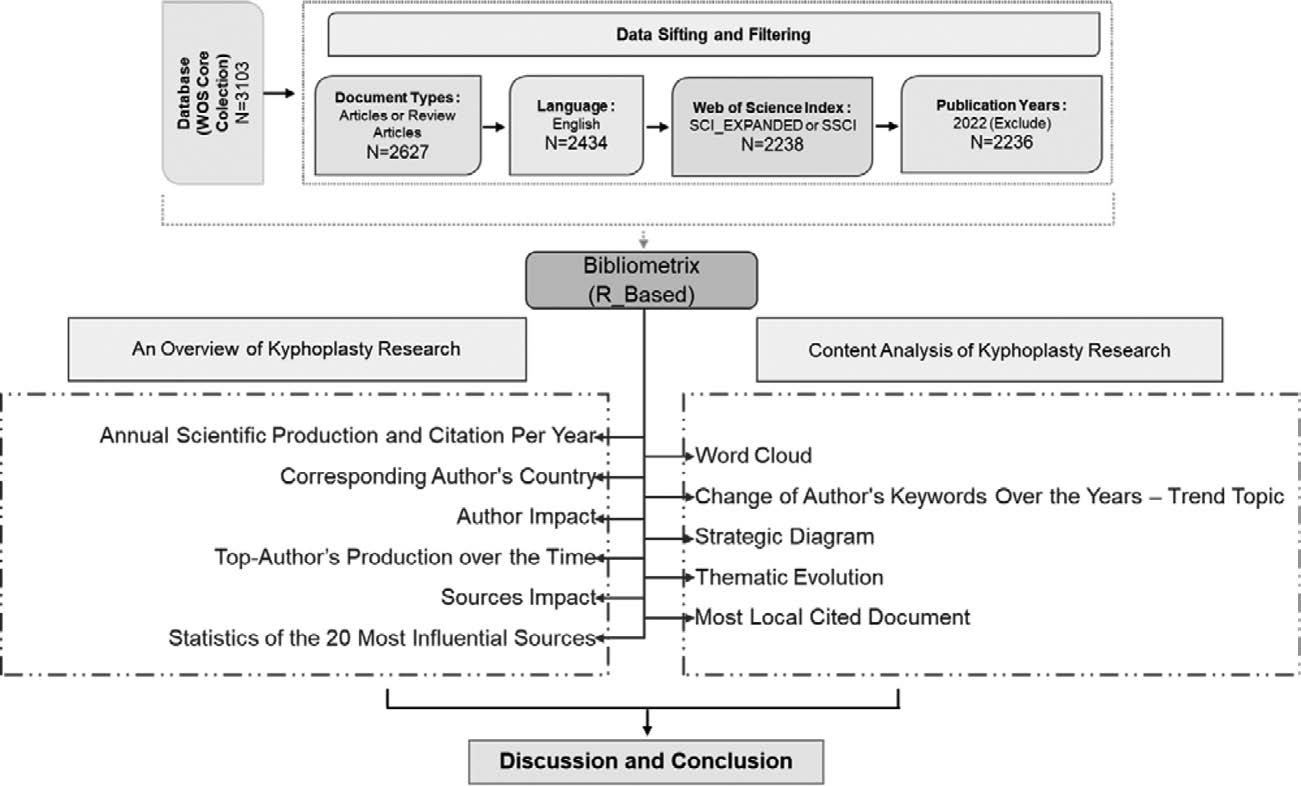
Workflow of science mapping.

The research framework of the Kyphoplasty study is presented in Figure 1. To conduct the bibliometric analysis of the subject of Kyphoplasty, the Web of Science Core Collection database that contains qualified data was preferred. Web of Science is considered one of the most comprehensive and qualified databases.^[9]^

After the selection of the data set, the data were filtered. The WoS database was searched on December 24, 2021. As a result of the search performed on Kyphoplasty in the database, 3103 articles were accessed. Only “Articles and Review Articles” were selected to extract irrelevant literature information, and the number of articles obtained decreased to 2627. When only “English” was chosen as the publication language, articles decreased to 2434. When “SCI_EXPANDED” and “SSCI” were selected as indexes, the number of articles became 2238. Finally, since the publication processes of the articles continued in 2022 in the current situation, the articles of 2022 were excluded from the review. As a result, the study was carried out on 2236 articles.

### 2.2. Bibliometric analysis

Bibliometrics is open-source software written in R and was preferred to conduct the bibliometric analysis of the data obtained from the WoS database since it is one of the most commonly used software for scientific literature mapping.^[6]^

### 2.3. General analysis of articles

The reviewed articles were analyzed in two parts. Researchers, journals, articles, institutions, and the studies’ countries were examined first. After this examination, *h*, *g*, and *m* indices were calculated. In the second part, the intellectual structure of the subject of Kyphoplasty was analyzed through content analysis. To perform a content analysis of the Kyphoplasty subject, word and citation analyses were utilized. Moreover, the intellectual structure analysis was performed to investigate the studies’ focus, main topics, and thematic development levels.

In 2005 Jorge Hirsch theorized the H-index or Hirsch index to calculate the citation index of an author. The main idea behind the h-index is that, for example, if an author has X articles cited at least X times by other authors, the author’s h-index is equal to X.^[10]^

Leo Egghe created the G-index in 2006. The H-index does not use the average number of citations to measure the citation performance of an article. Therefore, the G-index was developed to address this shortcoming. Unlike the H-index, articles with many citations are considered more in the G-index.^[11]^

One of the criticisms about the h-index is that when evaluating the academic success of young scientists, the fact that their articles do not have enough time to be cited is not taken into account. To assess the academic performance of young scientists, the impact factor can be recognized as a better candidate. Moreover, one way to compare academic histories of different lengths is to compare the h-index to years of active academic study. Like the h-index, Hirsch also developed the m-index.^[12]^

Within the scope of content analysis, a word cloud was produced for the keywords of the articles on Kyphoplasty using Bibliometrics. The word cloud is a graphical visualization of current headlines on Kyphoplasty. With the help of the word cloud, different association areas and the most dominant terms can be determined.^[13]^

### 2.4. Content analysis of articles

#### 2.4.1. Thematic mapping.

Thematic mapping reveals the temporal dynamics of research areas. The thematic development of Kyphoplasty research covering 2001 to 2021 was analyzed from a dynamic perspective. The research period (2001–2021) was divided into four sub-periods. The first sub-period was determined as ten years (2001–2010) due to the limited number of publications during the early years. The second sub-term is determined as 5 years (2011–2015), the third sub-term is determined as 3 years (2016–2018), and the fourth sub-term is determined as 3 years (2019–2021).

Thematic mapping was performed with the help of word analysis in the Bibliometrix program, and strategic diagrams were produced. For the word analysis, keywords were used since the authors determined the keywords and chose the words that best represent the article. Therefore, since the keywords represent the article, the dynamics of the study subject can be determined by performing keyword analysis.^[14]^

A strategic diagram reflects the interaction of actors in a research field over a while; with this aspect, it is a static description of the network structure of a scientific field. By analyzing the temporal dynamics of various configurations or interactions between networks in the same period, variables can be revealed deeper.^[15]^

The most repeated keywords are shown as theme clusters in the word analysis. For this purpose, the first 250 keywords were used in the analysis. The words in the clusters are the keywords with the highest frequency of use in the cluster. The size of the clusters reflects the frequency of use of keywords. The sub-periods were divided into four. Each theme cluster period shows different themes, and two criteria were used to scale the periods: centrality and density. Density demonstrates the *y*-axis of the thematic map, and centrality demonstrates the *x*-axis. The centrality grades the importance of the chosen theme and intensity development.^[16–18]^

• “Motor Themes” is the first quarter theme. It forms the upper right part, which denotes high density and centrality. Keywords in this theme have strong interlinking.^[18]^
• “Highly Developed and Isolated Themes” is the second quarter theme that expresses higher density and lower centrality. It forms the upper left part. They are essential for developing the subject of study.^[18]^
• “Emerging or Declining Themes” is the third quarter theme. It forms the lower left part. They are emerging or diminishing themes. It is a low centrality and intensity theme.^[18]^
• “Basic and Transversal Themes” is the fourth quarter theme. It forms the lower right part. It has a low density and high centrality. The keywords in this theme have strong interconnections. This theme includes frequently repeated words and has strong associations.^[18]^

### 2.5. Thematic evolution map

The Sankey Diagram was utilized to construct the thematic evolution map. In this diagram, each connection point represents a set of themes. The size of the nodes is directly proportional to the number of keywords. Flow lines between nodes point to the direction of the evolution of theme clusters over time. The edge width of the node is the sum of the connected elements.^[19]^

### 2.6. Citation analysis

The development dynamics of research topics can be examined by conducting citation analysis. In our study, citation analysis was performed to analyze the articles on Kyphoplasty and their relationships. Local Citations can be defined as the number of citations cited by the articles in the dataset. The Global Citation shows the number of citations to the article from the WoS database. It is necessary to eliminate the effect of publication date from citation analysis because newly published articles do not have enough time to be cited. Annual Local Citations (LC/YYP) and Annual Global Citations (GC/YYP) parameters eliminate this effect.^[20]^

### 2.7. Ethics

No analysis was performed on humans or animals in our study. Therefore, ethics committee approval is not required for our research.

## 3. Results

### 3.1. Overview

The first study on the subject of Kyphoplasty was conducted in 2001. The number of authors examined in 2236 articles is 7798, and the number of studies with a single author is found to be 56. While the number of studies per author is 0.287, the number of authors per study is determined as 3.49, and the collaboration index is 3.57.

### 3.2. Annual scientific production and citation per year

**Figure 2.**
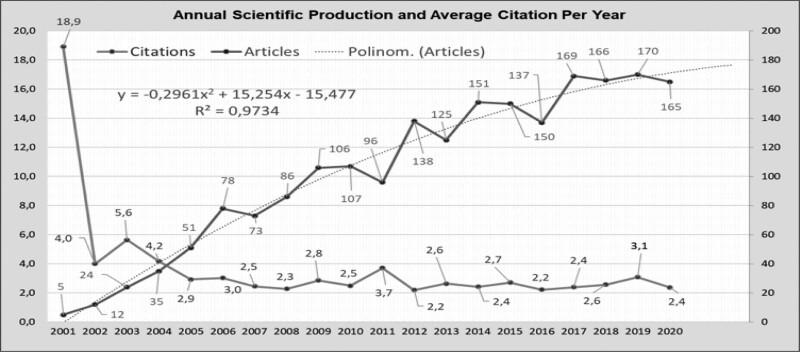
Annual scientific production and citation per year.

Annual scientific production and annual average citations for Kyphoplasty between 2001 and 2020 were obtained using Bibliometrix, as shown in Figure 2. Since the analysis program considers 2021 as an ongoing year, 166 articles belonging to 2021 are not included in this graph. It is seen that the number of articles in the field of Kyphoplasty increased from 2001 to 2020. While the annual number of articles was five in 2001, it reached 165 in 2020. Most publications were made in 2019. Our study added the trend line, the trend line equation, and the *R*^2^-confidence coefficient to the annual scientific production numbers graph. It can be said that the closer the confidence coefficient value is to integer 1, the stronger the trend line added to the chart represents the data. The confidence coefficient value we obtained was very close to 1 with 0.97 and represented the received data at an adequate level. The equation representing the trend line is presented on the graph.

The highest annual citation amount was reached when the average citations per article were examined, with 18.9 of the articles published in 2001. The annual average number of citations was 5.6 in 2003 and 3.1 in 2019. It is seen that the annual average citations maintained a certain level over the years. It can be said that the number of citations of the publications in recent years is significant, especially since more recent publications require time to be cited.

### 3.3. Country statistics

Kyphoplasty studies were analyzed in terms of countries, and the top 20 countries are presented in Table 1. The table is given in order of complete publication. According to the number of articles published in Kyphoplasty, China, the USA, Germany, Korea, and Italy are the leading countries.

**Table 1:** Top 20 countries in Kyphoplasty research.

Country	TPC	SCP	MCP	MCP_Ratio
China	701	667	34	0.0485
USA	515	441	74	0.1437
Germany	226	185	41	0.1814
Korea	97	96	1	0.0103
Italy	91	72	19	0.2088
France	81	72	9	0.1111
UK	69	49	20	0.2899
Japan	57	52	5	0.0877
Switzerland	51	29	22	0.4314
Turkey	44	40	4	0.0909
Spain	40	27	13	0.325
Greece	39	24	15	0.3846
Netherlands	31	24	7	0.2258
Canada	27	19	8	0.2963
Sweden	20	12	8	0.4
Australia	19	5	14	0.7368
Austria	18	13	5	0.2778
Israel	13	11	2	0.1538
Belgium	12	8	4	0.3333
Poland	12	12	0	0

MCP = Multiple country publications, SCP = Single country publications, TPC = Total number of publications by the corresponding author’s country.

While China ranks first with 667 publications in single-country publications and the USA ranks second with 441 publications, the USA ranks first with 74 publications in multi-country publications, and China ranks second with 34 publications. The country with the highest Multiple country publications (MCP) ratio, defined as the division of multi-country publications by the total publication number, is Australia, with a value of 0.7368.

### 3.4. Author statistics

The statistics of the top 20 authors who carried out the most impactful studies on Kyphoplasty between 2001 and 2021 are presented in Table 2. The table was produced according to the h-index. In Table 2, in the order of the author; the h-index, g-index, m-index, total citations, the total number of publications, and first year of publication (PY-baseline) in the field of Kyphoplasty were analyzed.

**Table 2:** Statistics of the 20 most influential authors in the field of Kyphoplasty research.

Author	h_index	g_index	m_index	TC	NP	PY_start
Yang HL	20	28	1.333	1178	86	2007
Hirsch JA	15	21	0.938	477	26	2006
Lieberman IH	14	19	0.667	1544	19	2001
Masala S	14	22	0.737	496	23	2003
Pflugmacher R	14	24	0.875	754	24	2006
Simonetti G	12	15	0.632	320	15	2003
Chen L	11	15	0.733	383	15	2007
Heini PF	11	12	0.611	434	12	2004
Kallmes DF	11	17	0.733	490	17	2007
Kasperk C	11	14	0.647	701	14	2005
Lane JM	10	12	0.5	427	12	2002
Laufer I	10	14	0.769	760	14	2009
Massari F	10	12	0.526	266	12	2003
Meeder PJ	10	12	0.588	593	12	2005
Zou J	10	16	0.833	277	19	2010
Anselmetti GC	9	10	0.6	324	10	2007
Bilsky MH	9	13	0.643	681	13	2008
Boszczyk BM	9	13	0.474	350	13	2003
Fuentes S	9	15	0.6	262	15	2007
Lis E	9	13	0.692	688	13	2009

NP = Total Number of Publications, PY_start = Publication year starting, TC = Total Citations.

According to the authors of the articles, the authors with the highest h-index value are Yang HL (20) and Hirsch JA (15). The authors with the highest G index are Yang HL (28) and Pflugmacher R (24). The author with the highest m-index is Yang HL (1.3), and the author with the second-highest m-index is Hirsch JA (0.938). The author with the highest number of citations is Lieberman IH, and the author with the highest number of articles is Yang HL. Zou J, who published an article for the first time in 2010, draws attention to its h-index (10), g-index (16), m-index (0.833), and total number of citations (277), and total number of publications (19).

**Figure 3.**
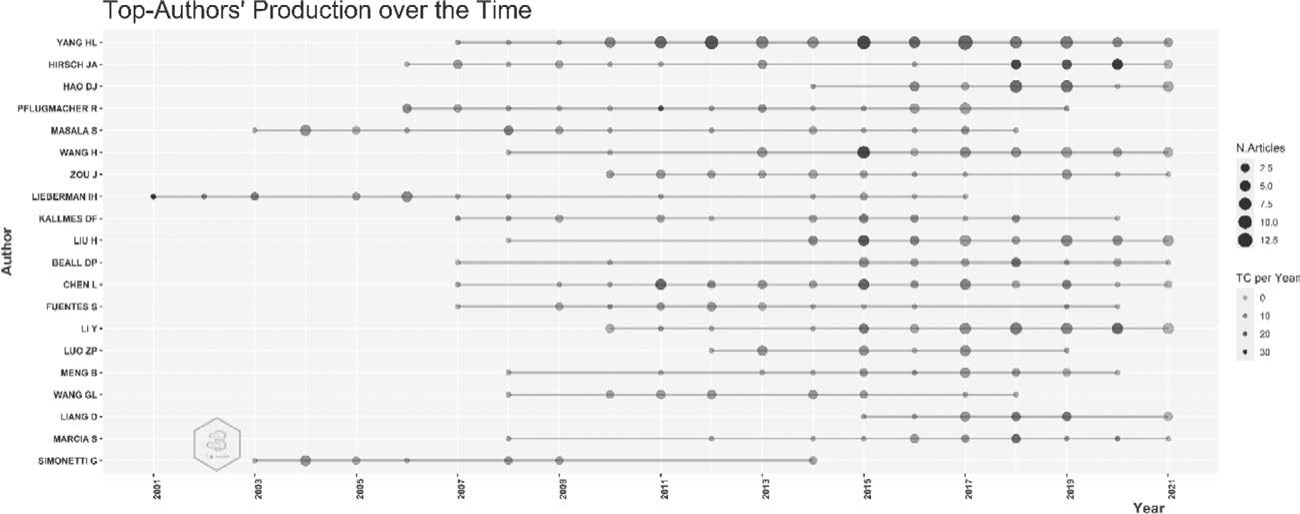
Production of top authors over time.

Figure 3 shows the performance of the authors who published articles about Kyphoplasty over time. Considering the length of the article’s publication line, the author who published on Kyphoplasty for the longest time is Lieberman (2001–2017). Hirsch JA, who published an article for the first time in 2006, continues his academic career. The size of the circles in the figure indicates the excess number of articles in that year. Yang HL published 13 articles in 2017; therefore, Yang HL is the most published author on the Kyphoplasty study in a year. The darkness of the circles in the figure indicates the total amount of citations the author has received per year. Hirsch JA reached the highest number of citations in 2020, with 28.5 citations. The fact that the author has been highly cited in recent years shows that he is effective in Kyphoplasty. 17 out of the 20 authors started working in Kyphoplasty in 2006 and later.

### 3.5. Journal statistics

**Figure 4. 20:**
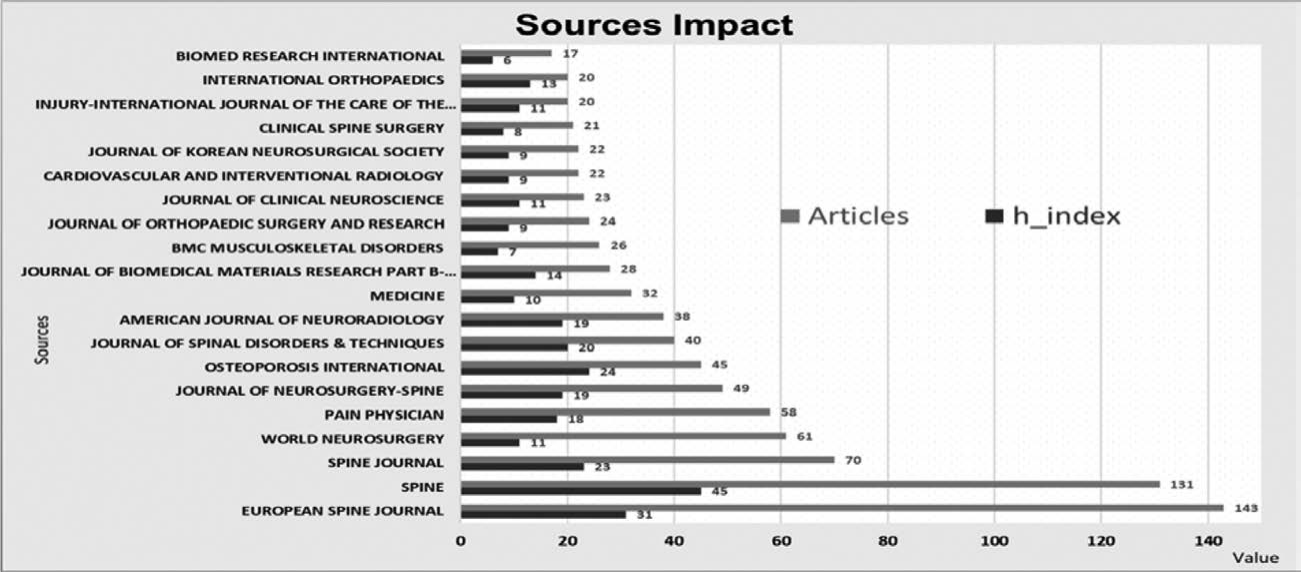
Most influential journals on Kyphoplasty.

Articles about Kyphoplasty have been published in a wide variety of journals. The 2236 articles analyzed were published in 380 different journals. The number of articles and the journal’s h-index were analyzed to identify the most influential journals in the Kyphoplasty research field. Figure 4 shows the 20 journals that have published the most articles on Kyphoplasty. The order in the figure is according to the number of publications. These 20 journals can be considered the most impactful resources in the field of Kyphoplasty. The *European Spine Journal* published 143 articles, and the *Spine* Journal published 131 articles. The journals with the highest H-index are Spine (45) and *European Spine Journal* (31). Although the number of articles published in the *Spine* journal is less than in other journals, it stands out with its high h-index (45).

Table 3 shows the top 20 journals according to the number of articles. 39.8% (890/2236) of the total articles were published in these journals. The *European Spine Journal* has the most publications, representing 6.39% (143/2236) of the total articles. Spine (7845) and *European Spine Journal* (3921) are the most cited Kyphoplasty journals.

**Table 3:** Statistics of 20 most influential journals in Kyphoplasty research.

Source	NP	TC	TC/NP	h_index	PY_start
*European Spine Journal*	143	3921	27.42	31	2003
*Spine*	131	7845	59.89	45	2001
*Spine Journal*	70	2108	30.11	23	2007
*World Neurosurgery*	61	540	8.85	11	2010
*Pain Physician*	58	910	15.69	18	2008
*Journal of neurosurgery-spine*	49	1118	22.82	19	2005
*Osteoporosis International*	45	1860	41.33	24	2001
*Journal of Spinal Disorders & Techniques*	40	1196	29.90	20	2004
*American Journal of Neuroradiology*	38	1204	31.68	19	2001
*Medicine*	32	287	8.97	10	2016
*J. of Biomed. Materials Res. Part B-Applied Biomaterials*	28	686	24.50	14	2006
*BMC Musculoskeletal Disorders*	26	205	7.88	7	2011
*Journal of Orthopedic Surgery and Research*	24	242	10.08	9	2010
*Journal of Clinical Neuroscience*	23	404	17.57	11	2007
*Cardiovascular and Interventional Radiology*	22	400	18.18	9	2006
*Journal of Korean Neurosurgical Society*	22	200	9.09	9	2007
*Clinical spine surgery*	21	182	8.67	8	2016
*Injury-Int. J. of The Care of The Injured*	20	515	25.75	11	2005
*International Orthopedics*	20	402	20.10	13	2006
*Biomed Research International*	17	117	6.88	6	2014

NP = Total Number of Publications, PY_start = Publication year starting, TC = Total Citations.

In addition, the number of citations per article, which shows the ratio between the number of citations and the number of articles for each journal, was also analyzed. Spine offers the highest value, averaging 59.89 citations per article. When the h index (10), the number of citations (287), and the number of publications (32) are evaluated, it is possible to say that the Journal of Medicine, first published in 2016, became very effective in the field of Kyphoplasty in a relatively short time.

### 3.6. Content analysis

In this section, keyword analysis and citation analysis were performed using bibliometric methods to identify the main elements of the Kyphoplasty research subject.

### 3.7. Frequency analysis for keywords

**Figure 5.**
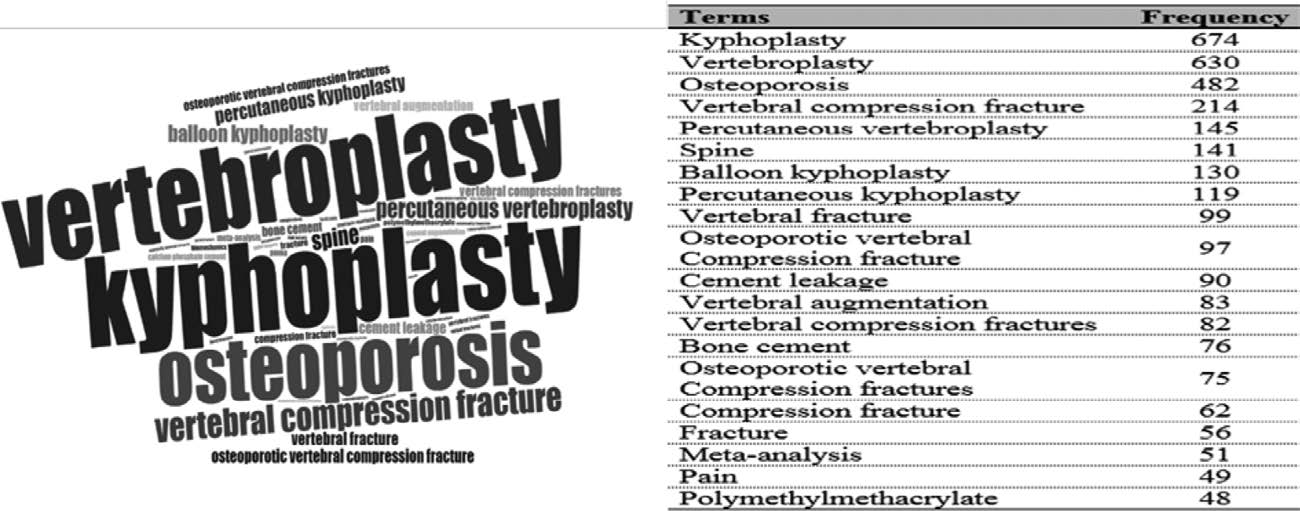
Word cloud and number of repetitions of keywords in the Kyphoplasty research area.

Bibliometrics was used to obtain data on the keyword frequency (repeat count) of the Kyphoplasty research subject, and the data obtained are presented in Figure 5. The word cloud is a graphical display of the latest topics in the Kyphoplasty research area. In Figure 5, the top 20 emerging keywords preferred by the authors are visualized. The size of the keywords in the image is directly proportional to the frequency of their appearance in the data set. The most used keywords are “kyphoplasty” and “vertebroplasty.” However, keywords such as “osteoporosis,” “vertebral compression fracture,” “percutaneous vertebroplasty,” and “spine” draw attention to the word cloud. The number of usages of the 20 most used keywords can be seen on the right side of the figure.

### 3.8. Keywords trend analysis

**Figure 6.**
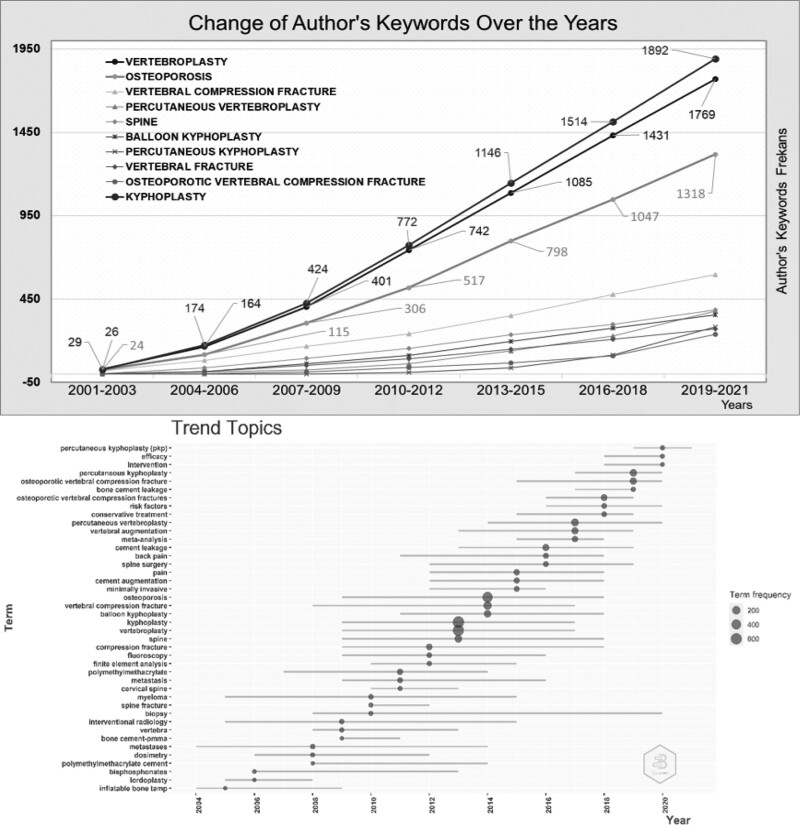
Change and orientation of the keyword in the field of Kyphoplasty by keyword frequency.

The change and trend of author keywords over time are presented in Figure 6. To analyze the subject of Kyphoplasty in different periods, the time interval from 2001 to 2021 is divided into seven equal time zones. The first ten author keywords of the articles were examined. From 2001 to 2021, the usage amount of all keywords increased. While the word Kyphoplasty was used 26 times in 2001 to 2003, it was used 1892 times in 2019 to 2021. The keywords vertebroplasty, osteoporosis, and vertebral compression fracture have been increasingly used together with the word Kyphoplasty. The keywords percutaneous vertebroplasty and percutaneous Kyphoplasty have increased significantly in recent years. The upper graph in Figure 6 shows the change in the usage amount of 10 keywords over time, and the lower graph shows the years in which the keywords are used frequently. In Kyphoplasty, the keywords inflatable bone tamp, pyloroplasty, and bisphosphonates were used more frequently in the 2000s. In contrast, percutaneous kyphoplasty, efficacy, and intervention have been used more frequently in the present day. The keywords kyphoplasty and vertebroplasty reached the highest number of uses in 2013 and osteoporosis in 2014.

### 3.9. Thematic development analysis

**Figure 7.**
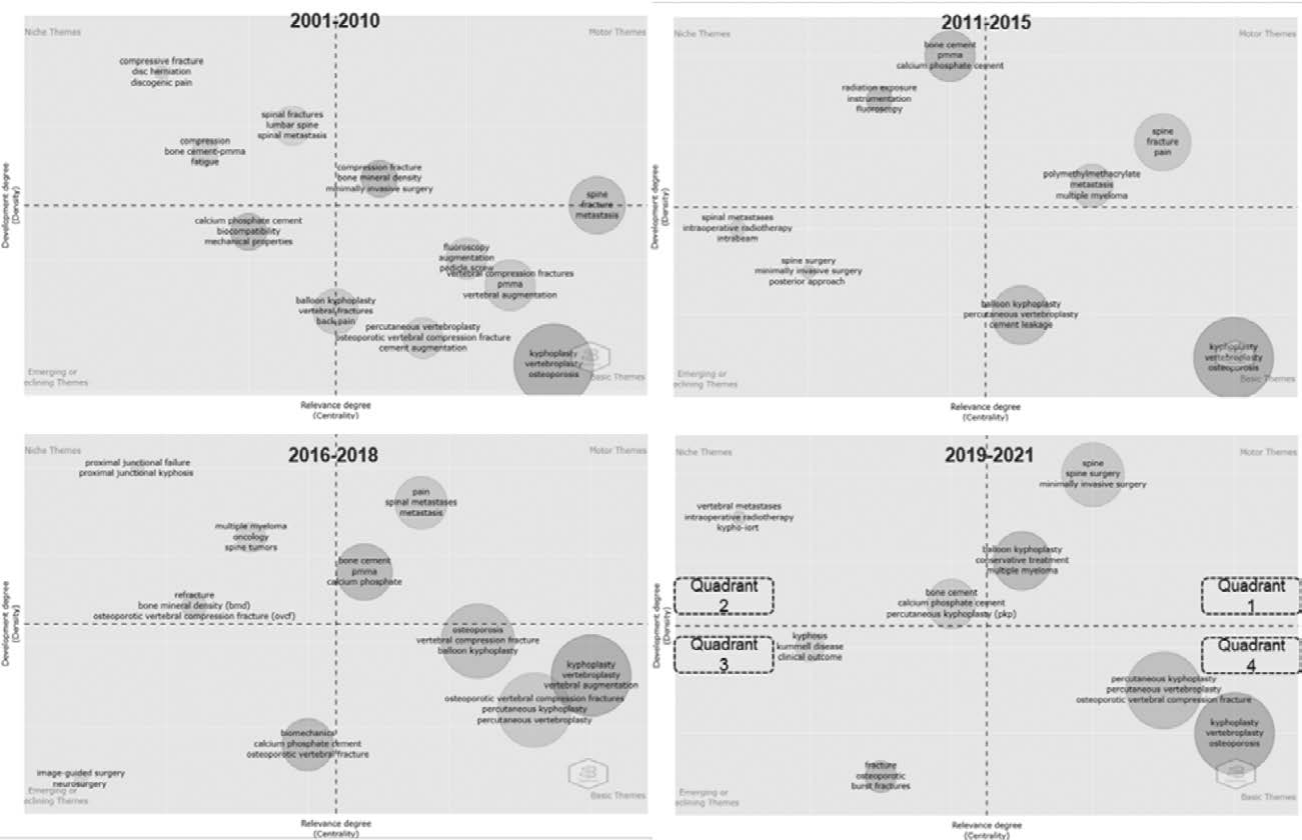
Strategic diagram of Kyphoplasty studies (2001–2021).

Strategic diagrams of the Kyphoplasty research subject are presented in Figure 7.

“Motor Themes” is the 1st quarter theme presented at the top right. In the 2019 to 2021 period, the first cluster was represented by the keywords “spine, spine surgery, minimally invasive surgery,” and the second cluster represented “balloon kyphoplasty, conservative treatment, multiple myeloma.”

“Highly Developed and Isolated Themes” is the 2nd quarter theme at the top left. In the 2019 to 2021 period, the first cluster was represented by the keywords “bone cement, calcium phosphate cement, percutaneous kyphoplasty,” The second cluster was represented by the keywords “vertebral metastases, intraoperative radiotherapy, kyphotic-iort.”

“Emerging or Declining Themes” is the 3rd quarter theme in the lower-left part of the thematic map. In 2019 to 2021, the keywords “fracture, osteoporotic, burst fractures” represented the first cluster, and “kyphosis, kummell disease, clinical outcome” represented the second cluster.

“Basic and Transversal Themes” is the 4th quarter theme presented in the lower right part of the thematic map. In the 2019 to 2021 period, the keywords “kyphoplasty, vertebroplasty, osteoporosis” represented the first cluster, and “percutaneous kyphoplasty, percutaneous vertebroplasty, osteoporotic vertebral compression fracture” represented the second cluster.

**Figure 8.**
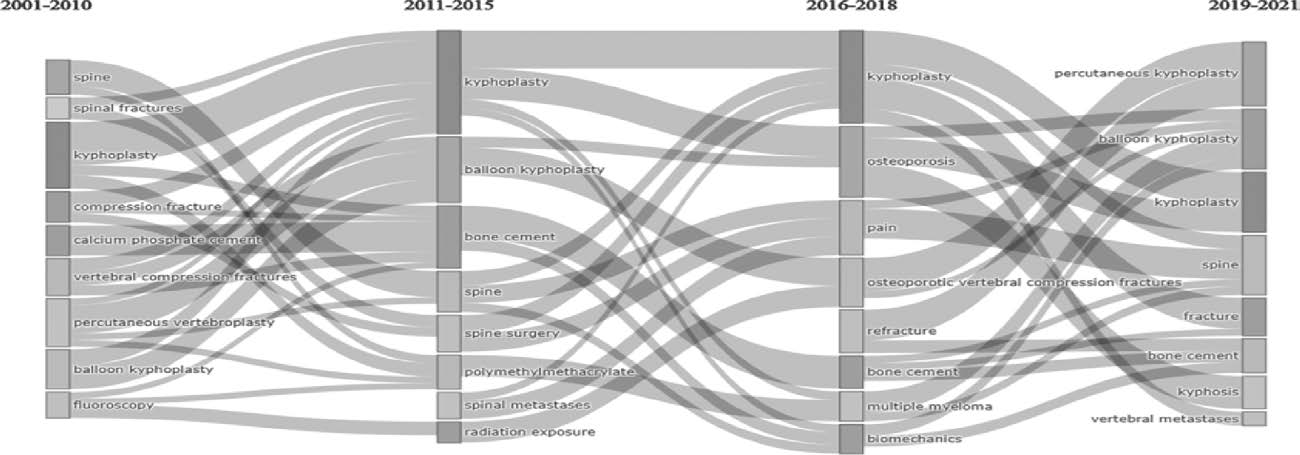
Thematic development of Kyphoplasty research (2001–2021).

In addition to the 4-term Thematic Map, the four-term thematic evolution mapping presented in Figure 8 was used to evaluate the development and change of Kyphoplasty themes over the years.

The principles in Figure 7 were used while producing the Thematic Evolution Map in Bibliometrics. When the diagram is examined, it can be seen that there are nine themes in the first period, eight themes in the second period, eight themes in the third period, and eight themes in the last period. Since the node’s width is proportional to the size of the linked keywords, it can be seen that Kyphoplasty, which is found in every period, fed more keywords and was fed by more keywords in 2011 to 2015. While it was fed by the keywords “spine, kyphoplasty, compression fracture, vertebral compression fracture, percutaneous vertebroplasty, balloon kyphoplasty” in the 2011 to 2015 period, it fed the keywords “kyphoplasty, osteoporosis, multiple myeloma, biomechanics.” In the 2019 to 2021 period, the keywords “percutaneous kyphoplasty, balloon kyphoplasty, spine, fracture, bone cement, kyphosis, vertebral metastases” and the keyword “kyphoplasty” matured in the lower periods and took place as a theme.

### 3.10. Citation analysis

Table 4 lists the 20 most cited articles on the Kyphoplasty according to their local citation values. The article “Garfin SR, 2001” is the most useful in Kyphoplasty since it obtained 490 local and 740 general citations. The most cited article in recent years belongs to “Klazen CAH, 2010” with local citation (286) and global citations (GC) (539) values. (LC/YYP) and (GC/YYP) concepts have been utilized to eliminate the disadvantage caused by the limited time of the articles published in recent years to obtain citations. The article with the highest LC/YYP and GC/YYP values is “Klazen CAH, 2010”.

**Table 4:** Top 20 local articles on Kyphoplasty.

Document	YP	LC	LC/YYP %	GC	GC/YYP %	LC/GC ratio %
Garfin SR, 2001, Spine	2001	490	23.333	740	35.238	66.22
Lieberman IH, 2001, Spine	2001	429	20.429	664	31.619	64.61
Hulme PA, 2006, Spine	2006	319	19.938	491	30.688	64.97
Wardlaw D, 2009, Lancet	2009	297	22.846	525	40.385	56.57
Klazen CAH, 2010, Lancet	2010	286	23.833	539	44.917	53.06
Fourney DR, 2003, J Neurosurg	2003	231	12.158	468	24.632	49.36
Fribourg D, 2004, Spine	2004	178	9.889	279	15.500	63.80
Eck JC, 2008, SPINE	2008	158	11.286	230	16.429	68.70
Dudeney S, 2002, J Clin Oncol	2002	145	7.250	247	12.350	58.70
Taylor RS, 2006, Spine	2006	142	8.875	226	14.125	62.83
Phillips FM, 2002, Spine	2002	140	7.000	216	10.800	64.81
Phillips FM, 2003, Spine-a	2003	140	7.368	192	10.105	72.92
Watts NB, 2001, Osteoporosis Int	2001	135	6.429	242	11.524	55.79
Ledlie JT, 2003, J Neurosurg	2003	130	6.842	191	10.053	68.06
Taylor RS, 2007, Eur Spine J	2007	128	8.533	190	12.667	67.37
Berenson J, 2011, Lancet Oncol	2011	127	11.545	274	24.909	46.35
Belkoff SM, 2001, Spine	2001	124	5.905	196	9.333	63.27
Theodorou DJ, 2002, Clin Imag	2002	118	5.900	163	8.150	72.39
Voggenreiter G, 2005, Spine	2005	118	6.941	168	9.882	70.24
Polikeit A, 2003, Spine	2003	115	6.053	287	15.105	40.07

GC/YYP = Annual Global Citations, GC = Global Citations, LC/YYP = Annual Local Citations, LC = Local Citations, YP = Year of Publication, YYP = Year 2022-Year of Publication.

The article “Garfin SR, 2001” is the most influential in Kyphoplasty. Another concept developed for the most-cited authors is Local Citation Percentage. According to the Local Citation percentage, the most influential article is “Philips FM, 2003,” with 72.92%.

## 4. Discussion

The publication of articles on Kyphoplasty in journals coincides with the beginning of the 80s. An increase was observed in the number of articles published in the following years. In our study, countries were examined regarding the articles they published on Kyphoplasty. The USA, the world leader in the academic field, is behind China regarding articles on Kyphoplasty. The USA and Germany follow China. When all medical literature is considered, the USA, China, England, and Germany are the first places in the world. On Kyphoplasty, Korea and Italy are the fourth and fifth countries with the highest number of articles published. It is also noteworthy that Switzerland, which is not listed in the medical literature, is in the top ten regarding articles on Kyphoplasty. However, when multiple country publications with the participation of authors from different countries are examined, the most successful country is Australia, with the highest MCP/TCP value.

One of the fast-developing countries with strong potential in the medical tourism industry is South Korea, a model country in the medical tourism industry. It is possible that this potential of the country affected the number of procedures related to Kyphoplasty and thus the published articles.^[21]^

Switzerland is ahead of other countries in Kyphoplasty mainly because the treatment costs are cheaper than in other European countries. For this purpose, tourists go to Switzerland from European countries. In addition, private health services are prevalent and bring essential advantages to Switzerland in health tourism. Both cheap and high-quality health services have caused Kyphoplasty procedures to be performed more frequently in Switzerland and, therefore, more articles to be published in this country.^[22]^

It is seen that the top contributor to Kyphoplasty appears to be Professor Hui-Lin Yang, MD, Ph.D. He is president of the Clinical Research Institute of Soochow University and chief of the Institute of Orthopedics department. His specialty is orthopedic surgery. He is more successful than other authors in the h and g indexes. After Professor Hui-Lin Yang, the researcher with the highest h index is Joshua Hirsch. Joshua Hirsch MD is Vice-Chair Procedural Services and Service Line Chief of Neuro Interventional Radiology at Massachusetts General Hospital.

On the other hand, the most cited author is Isador H. Lieberman. Dr Isador H. Lieberman is a fellowship-trained Orthopedic and Spinal Surgeon. He is board certified by the American Board of Orthopedic Surgery. Dr Lieberman specializes in the surgical treatment of spinal disorders at the Texas Back Institute. The most important factor underlying the success of Dr Lieberman is that he started his academic life earlier than other scientists. While a significant part of other scientists published their articles for the first time in 2006, and later, Dr Lieberman started to publish in 2001. Many of the most influential researchers published their first papers in 2006. This is because Kyphoplasty is a new technique. Phase studies of the technique started in 2001. The first studies are also from this date, and one of the first studies was published by Dr Lieberman.^[23]^

Jun Zou, MD, Ph.D. is an associate professor Department of Orthopedics Surgery at Affiliated Hospital of Soochow, China. Jun Zou and Professor Hui-Lin Yang are Chinese and work at the same institution. Jun Zou published an article for the first time in 2010. He is the youngest of the authors. This situation is also reflected in the m-index values used to compare researchers who started to publish articles at different times. Jun Zou has one of the highest m-index values among other researchers.

The *European Spine Journal* is the journal that publishes the highest number of articles in Kyphoplasty. Spine and Spine Journal follow this. When considering the H-index value, Spine is ahead of the others. The number of citations it receives according to the number of articles is very high compared to other journals. This is reflected in the citations per paper (TC/NP) value. The TC/NP value of the Spine journal is close to twice that of the closest journal. According to the number of articles, the Osteoporosis International journal in the seventh-place ranks third among 20 journals with an h-index score of 24. Like the Spine, Osteoporosis International has a very high TC/NP value.

On the contrary, *World Neurosurgery* and *Pain Physician* journals have fewer citations, although they have published more articles than other journals. This situation was reflected in the TC/NP value of both journals. *European Spine Journal* is the official publication of The Spine Society of Europe. The Spine Journal the official journal of the North American Spine Society.

According to the analysis results, “kyphoplasty” and “vertebroplasty” are the most frequently used keywords. The words “osteoporosis,” “vertebral compression fracture,” and “percutaneous vertebroplasty” are also frequently used. These keywords are all related to the procedure. For this reason, it is an expected result that the keywords are used most frequently.

Polymethylmethacrylate draws attention among the keywords. In the Kyphoplasty and Vertebroplasty procedure, polymethylmethacrylate is the substance that reconstructs the vertebra by being injected into the vertebral body. It is more commonly referred to as “Cement.” Both “cement” and “polymethylmethacrylate” are among the most frequently used keywords.^[24]^

When the strategic diagrams on Kyphoplasty are examined, one of the words that have come to the fore in recent years within the motor theme is “minimally invasive surgery.” Kyphoplasty is a minimally invasive surgical procedure developed to treat vertebral compression fractures due to osteoporosis. Therefore, a minimally invasive surgery keyword has been used frequently.

One word that requires attention in the motor theme is “multiple myeloma.” Myeloma bone disease is one of the complications of multiple myeloma. More than 80% of myeloma patients develop destructive bone lesions. One of the most frequently involved areas is the vertebrae, which have prevalent fractures. The kyphoplasty procedure is also used in the treatment of fractures. This has highlighted multiple myeloma among the keywords.^[25,26]^

In recent years, calcium phosphate and Kypho-IORT have come to the fore in Highly Developed and Isolated Themes. Calcium phosphate cement is a relatively new filling material utilized in Kyphoplasty applications. It is rapid hardening, mechanical durability, and biocompatibility properties stand out. It can be an alternative to polymethylmethacrylate, which is the gold standard. Kyphoplasty is used in the treatment of bone metastases of cancers. However, Kyphoplasty alone does not have an anti-cancer effect. New techniques have been developed, combined with (physical) anti-cancer methods such as Kyphoplasty. Kypho-IORT is the combination of Kyphoplasty and intraoperative radiotherapy.^[27,28]^

One of the main keywords in Emerging or Declining Themes is “Kummell disease.” Kummell disease is a specific osteoporotic vertebral compression fracture that develops in the thoracolumbar spine segment. It is known as intravertebral avascular osteonecrosis. Kyphoplasty and percutaneous vertebroplasty are widely used to treat Kummell disease.^[29]^

The thematic development of Kyphoplasty research was examined through thematic development analysis. The dynamics of the keywords in the strategic diagrams in each sub-period are given in the paragraphs above.

The article “Kyphoplasty and Vertebroplasty for the Treatment of Painful Osteoporotic Compression Fractures,” published by Garfin SR in Spine journal in 2001, is the most influential article on Kyphoplasty. It is thought that the article’s subject, that is, the techniques that come into use, and its publication in an impactful journal such as Spine, affects the number of citations.^[30]^

The article “Vertebroplasty versus conservative treatment in acute osteoporotic vertebral compression fractures (Vertos II): an open-label randomized trial” published by Klazen CAH in the Lancet journal in 2010 is the most cited article among the articles published in recent years. This article compares vertebroplasty with traditional treatments for compression fractures of the vertebrae. It was investigated whether vertebroplasty has additional significance compared to optimal pain treatment.^[31]^
